# Development of a point-of-care field test for Tasmanian devil facial tumour disease

**DOI:** 10.1098/rsos.260028

**Published:** 2026-11-11

**Authors:** Anuk Kruawan, Rajendra KC, David A. Gell, Jocelyn M. Darby, Weizhen Zhu, Chrissie E. B. Ong, Kirsten A. Fairfax, Andy Flies

**Affiliations:** ^1^ College of Health and Medicine, University of Tasmania Menzies Institute for Medical Research, Hobart, Australia; ^2^ Tasmanian School of Medicine, University of Tasmania, Hobart, Australia

**Keywords:** Tasmanian devil, CRISPR, DFT1, DFT2, DFTD, SHERLOCK, diagnostics, RPA, isothermal amplification, transmissible cancer

## Abstract

The Tasmanian devil (*Sarcophilus harrisii*) population has undergone a major decline in the wild owing to the epidemics of two transmissible cancers known as devil facial tumour 1 (DFT1) and devil facial tumour 2 (DFT2). Understanding the distribution and prevalence of DFT1 and DFT2 is challenging as they can only be detected in field conditions when tumours are large enough to be visually observed. This impedes our ability to understand how the devil population is evolving in response to the tumours. PCR-based diagnostics are available, but the technique is not field-applicable. To overcome these hurdles, we developed an ultrasensitive and field-deployable rapid diagnostic tool. We used a technique called specific high-sensitivity enzymatic reporter unlocking (SHERLOCK), which combines an isothermal nucleic acid amplification step with CRISPR-based recognition and activation of reporter probes to indicate target sequence detection. This system requires only portable laboratory equipment, making it more amenable to field conditions. The results showed that our test can detect DFT1 and DFT2 DNA within the attomolar range. We then confirmed that the test can detect DFT1 in the field using non-invasive swab samples. The SHERLOCK test can be used for more active monitoring and management approaches that are not currently possible.

## 1. Introduction

The Tasmanian devil (*Sarcophilus harrisii*) is the largest extant carnivorous marsupial and is found exclusively in the island state of Tasmania, Australia [1]. Devils became extinct on mainland Australia around 3000 years ago, possibly owing to competition with dingoes (*Canis lupus dingo*), climate change and human activity [2,3]. Devils inhabit various regions across Tasmania, including woodlands, eucalyptus forests and coastal areas [4]. Adult male devils typically weigh between 7.5 and 13 kg, while females range from 4.5 to 9 kg [1,5]. Both sexes can live at least 6 years in the wild [1,5]. Tasmanian devils are nocturnal and solitary scavengers, with their home ranges between 12 and 18 km^2^ [6]. Unfortunately, the wild devil population has greatly declined owing to two contagious cancers known as devil facial tumour 1 (DFT1) and devil facial tumour 2 (DFT2) [7,8].

DFT1 and DFT2 appear grossly similar, but they are genetically, histologically and cytogenetically different [8,9]. Various diagnostic assays are available to identify and distinguish the two tumour types, with ‘Tasman PCR’ being the gold standard [10]. While the Tasman PCR can effectively identify DFT1 and DFT2 DNA in tumour biopsies, the technique is challenging to implement in the field. This is owing to the requirement for specialized DNA extraction protocols or kits, a thermal cycler and other associated equipment. This can lead to diagnosis being made days or weeks after sample collection, limiting further investigation of suspected tumours when devils are in hand during trapping trips. Identification of devils that are exposed to DFT1 or DFT2, but do not develop visible tumours or exhibit natural tumour regressions is critical information for devil conservation management and understanding tumour and devil co-evolution [11–13].

Isothermal amplification assays, such as loop-mediated isothermal amplification (LAMP) and recombinase polymerase amplification (RPA), have been explored as alternative diagnostic methods for a variety of pathogens [14]. LAMP uses eight primers to efficiently amplify the target, while RPA uses two primers similar to PCR and can operate with PCR primers in some instances [15,16]. As LAMP requires more primers than RPA, the assay design and optimization is more challenging [17]. Studies have compared the amplification kinetics among various methods and found that RPA is more rapid and sensitive than LAMP or even PCR [18–20]. However, RPA usually suffers from non-specific amplification and primer dimerization owing to the lower amplification temperature of 37°C [20]. To improve the specificity of target detection, isothermal amplification techniques can be paired with clustered regularly interspaced short palindromic repeats (CRISPR) and CRISPR-Cas enzymes for secondary target sequence detection [14].

CRISPR evolved as a defence mechanism in prokaryotes to recognize and degrade pathogens' (i.e. bacteriophage) genetic material [21,22]. The programmable endonuclease activities have been harnessed for gene editing, namely the CRISPR-Cas9 system [23,24]. Furthermore, other types of Cas enzymes have been used as DNA and RNA detection tools. DNA endonuclease-targeted CRISPR trans reporter (DETECTR) [25] and specific high-sensitivity enzymatic reporter unlocking (SHERLOCK) [26] are key examples that use LbCas12a and LwCas13a enzymes with their corresponding programmable CRISPR RNA (crRNA) to detect DNA and RNA targets, respectively. These techniques incorporate RPA to pre-amplify the target before detection by CRISPR-Cas, which can achieve single-copy sensitivity with high specificity. DETECTR and SHERLOCK have been used to detect pathogens such as SARS-COV-2, *Plasmodium* spp. and foodborne diseases such as *Vibrio harveyias* [18,27,28]. Moreover, SHERLOCK was the first Food and Drug Administration (FDA)-approved CRISPR-based diagnostic tool used to detect SARS-COV-2 [29].

LbCas12a and LwCas13a recognize single-stranded (ss)DNA or ssRNA, respectively, and both enzymes exhibit collateral cleaving activity (i.e. cleavage of all DNA or RNA in the reaction) upon recognition of the target nucleic acid [25,26]. SsDNA and ssRNA reporters containing a quencher and fluorophore have been used to detect the collateral cleaving upon target recognition in LbCas12a and LwCas13a, respectively [25,26]. As LwCas13a only recognizes RNA targets, the pre-amplified DNA needs to be converted to RNA, usually through incorporation of a T7 promoter in the forward RPA primer and a T7 polymerase enzyme in the reaction mix, before the CRISPR-Cas cleaving can be activated [30]. In the case of RPA-CRISPR-Cas (SHERLOCK) diagnostics, all components only require a single incubation temperature, making it more amenable to field applications than conventional PCR or LAMP-CRISPR (LAMP operates at a higher temperature than CRISPR-Cas) [14].

Here, we describe the development of a SHERLOCK test requiring minimal instrumentation for field diagnosis of DFT1 and DFT2. Our SHERLOCK tests detect interchromosomal fusions identified in DFT1 and DFT2 [10]. The results showed that our test can detect DFT1 and DFT2 DNA within the attomolar DNA range. Pilot field testing revealed that our test could detect DFT1 with a high level of sensitivity and specificity and may have the potential to serve as a preclinical diagnostic tool for devil facial tumour disease (DFTD).

## 2. Material and methods

### 2.1. Cell lines and primary samples

DFT1 C5065 (RRID:CVCL_LB79) [31], DFT2 JV (RRID:CVCL_A1TN) and fibroblast cell lines (TD602 Rosie) were cultured in RPMI 1640 (Gibco, no. 11875093) supplemented with 10% heat-inactivated foetal bovine serum (CellSera, no. AU-FBS/PG), 50 µM 2-mercaptoethanol (Sigma-Aldrich, no. 28713-500G-F), 10 mM HEPES (Gibco, no. 15630080) and 1% Antibiotic-Antimycotic (Gibco, no. 15240062) at 37°C and 5% CO_2_ in a humidified incubator. Primary fibroblast cells were cultured with the same culture medium with added 10% AmnioMAX-II Complete Medium (Gibco, no. 11269016). Cells were harvested at 80% confluence for genomic DNA extraction using PureLink Genomic DNA Mini Kit (Invitrogen, no. K182001) according to the manufacturer's protocol.

Solid biopsies and swabs were taken from deceased and live devils. For deceased devils, swab samples of the superficial layer of tissue sites suspected of DFT1 or DFT2 were taken. A 0.5 cm^3^ solid biopsy was taken from the same tissue site using a 4 mm disposable biopsy punch (Kai Medical, no. EUR0450/10). This was conducted as part of the routine necropsies performed at the Tasmanian Museum and Art Gallery (TMAG). Swab and biopsy samples were either flash-frozen on dry ice for diagnosis in the laboratory or added to the SHERLOCK parasite rapid extraction protocol (S-PREP buffer) for field diagnosis. S-PREP formulation was obtained from [28]. The buffer consists of nuclease-free TE buffer (Invitrogen, no. AM9849) supplemented with 50 mM DTT and 20% (w v^−1^) Chelex-100 (Bio-Rad, no. 1432832). Additionally, non-DFT solid biopsy samples from two devils were included. These were anal sac tumour and round cell tumour. For live devils with ulcerated lesions, swab samples were taken and/or a biopsy was taken from the same spot using a 4 mm disposable biopsy punch (Kai Medical, no. EUR0450/10). Samples were taken from the area with or without cleaning with saline and gauze. If the tissue sites suspected of DFT1 or DFT2 were not ulcerated, either a swab or fine needle aspirate (FNA) samples were taken. Swab, biopsy and FNA samples were added to S-PREP buffer for downstream SHERLOCK diagnosis. Additionally, ear biopsies (0.5 cm^3^) were collected from wild devils and quolls similarly. Table S1 in the supplementary material shows a list of live devils and quolls that the samples were collected from alongside their clinical presentation.

As working with endangered species can raise ethical concerns such as animal welfare and ecological impact, research must minimize harm through non-invasive methods while strictly adhering to ethical guidelines. This ensures that our work supports scientific advancement and species protection. Therefore, all samples were collected with the approval of the University of Tasmania Animal Ethics Committee (permit number: A0026159), and the Department of Natural Resources and Environment (NRE) permit that protects threatened fauna used for scientific purposes (permit number: TFA22462).

### 2.2. Expression and purification of LwCas13a

Rosetta 2(DE3)pLysS Singles competent bacteria pre-transformed with pC013-Twinstrep-SUMO-huLwCas13a (Addgene no. 90097) were used as an expression system for LwCas13a. A detailed protocol is listed in the supplementary material, S1. The purified LwCas13a was diluted to 63.3 µg ml^−1^ in protein storage buffer for use with the CRISPR detection step. Protein fractions from each stage of purification were analysed using sodium dodecyl-sulfate polyacrylamide gel electrophoresis (SDS-PAGE) to ensure successful expression and purification. Briefly, 4 µl 10X Bolt sample reducing agent (Thermo Fisher Scientific, no. B0009), 10 µl 4X Bolt LDS Sample Buffer (Thermo Fisher Scientific, no. B0007), and 16 µl UltraPure water (Invitrogen, no. 10977015) were added to 10 µl of samples before being heated at 95°C for 5 minutes, then incubated on ice for 2 minutes. In total, 30 µl of samples were loaded into Bolt 4‐12% Bis-Tris Plus Mini Protein Gels (Thermo Fisher Scientific, no. NW04120BOX) in 1× Bolt MES SDS running buffer (Thermo Fisher Scientific, no. B0002). The gel was run at 200 V for approximately 20 minutes. The gel was stained for total protein using GelCode Blue Safe Protein Stain (Thermo Fisher Scientific, no. 24594) according to the manufacturer's protocol before being visualized (supplementary material, figure S1).

### 2.3. RPA primer design and screening

RPA primers were designed to amplify DFT1 and DFT2 markers, found on Chr2_GL841420:ChrX_GL867598 and Chr4_GL856969:Chr5_GL861630, respectively [10]. For DFT1, five forward primers and seven reverse primers were designed (supplementary material, table S2). For DFT2, five forward and five reverse primers were designed (supplementary material, table S2). Additionally, five forward and five reverse primers were designed for RPL13a to serve as an internal positive control (supplementary material, table S2). A region of the RPL13a sequence that was conserved between devil and brushtail possum (*Trichosurus vulpecula*) was selected, with the aim of developing RPA primers that could be used in other marsupial species.

All forward primers have a T7 RNA polymerase promoter sequence appended on the 5′ terminus (5′-gaaattaatacgactcactataggg-3′). Primers were designed as suggested by the manufacturer's RPA guidelines (TwistAmp Basic Kit, TwistDx). RPA primers specific for ZIKV were also included as a positive control for RPA and downstream CRISPR detection reaction [30] (supplementary material, table S2). All primers were synthesized by Integrated DNA Technologies (IDT). For primer screening, products from all primer combinations were extracted using NucleoSpin Gel and PCR Clean-up kit (Macherey-Nagel, no. 740609.250), then run on a 3% agarose gel. Top candidates were selected for further screening by coupling with SHERLOCK and various crRNAs, as described below.

### 2.4. RPA amplification

TwistAmp Basic Kit (TwistDx, no. TABAS03KIT) was used for our primer screening. The reaction mix was prepared for each set of primers. 0.56 µM of each primer (2.4 µl of 10 µM starting concentration) was added into 29.5 µl of TwistAmp rehydration buffer. A total of 8.65 µl of water was then added to the mixture before a quick vortex and spin-down, which resulted in 42.95 µl total volume. In total, 40 µl of the reaction mix was added directly into the RPA pellet. A total of 20 µl of the mixture was added into fresh PCR tubes (one reconstituted RPA pellet yields two RPA reactions). Unnecessary pipette tip changing was avoided, as a significant amount of reaction volume could be lost owing to the viscosity of the RPA reaction. In total, 2 µl of the sample in S-PREP buffer or gDNA (10 ng µl^−1^ starting concentration for primer screening) was added into corresponding RPA reactions. A total of 1.25 µl of magnesium acetate (MgOAc) provided with the TwistAmp Basic Kit (TwistDx) was then added to activate the reaction before the reaction was vortexed and centrifuged. The reaction was incubated at 37°C for 30 minutes (this was done as soon as possible, as the reaction starts the moment MgOAc is added). The post-amplification product was directly used for CRISPR-LwCas13a detection step.

### 2.5. S-PREP buffer preparation and crude DNA extraction

S-PREP formulation was obtained from [28]. The buffer consists of nuclease-free TE buffer (Invitrogen, no. AM9849) supplemented with 50 mM DTT and 20% (w v^−1^) Chelex-100 (Bio-Rad, no. 1432832). The strong chelating and reducing agents minimize the risk of enzymatic activity inhibition by inhibitors that are present in the crude lysate. Primary samples (from biopsy punches, fine needle aspirates, whole-blood samples, and swabs) were added to 500 µl S-PREP buffer in 1.5 ml microfuge tubes. Samples were then incubated at 95°C for 10 minutes to inactivate enzymes and enzymatic inhibitors and to release crude DNA from cells. The mixture was left to cool for 2 minutes on ice before being used in RPA amplification. The amount of solid and liquid biopsy should not exceed 1 mm^3^ and 100 µl, respectively.

### 2.6. CRISPR RNA (crRNA) design and screening

crRNAs were designed as suggested by Kellner *et al.* [30]. One crRNA was designed for each DFT1 and DFT2 (supplementary material, table S2). ZIKV crRNA sequence was obtained from Kellner *et al.* [30] (supplementary material, table S2). DFT1, DFT2 and ZIKV crRNAs were synthesized by IDT (Ultramer RNA Oligo, 4 nmol, standard desalting). For RPL13a, three crRNA DNA templates were designed for *in vitro* transcription (IVT; synthesized by IDT; supplementary material, table S2). The 10 µl annealing reaction for each crRNA consisted of 10 µM crRNA DNA template (1 µl of 100 µM stock), 10 µM T7-3G oligonucleotide (1 µl of 100 µM stock), 1X Standard Taq buffer (1 µl of 10X stock, NEB, no. B9014S), and 7 µl UltraPure water. The reaction was performed in a thermal cycler with a 5-minute denaturation at 95°C and cooled to 4°C using a 2.5% ramp rate. The annealed crRNA DNA templates were then added to the IVT reaction. A 40 µl reaction for each crRNA consisted of 10 µl annealed template, 5 mM NTP buffer mix (10 µl of 20 mM stock, HiScribe T7 Quick High Yield RNA Synthesis Kit, NEB, no. E2050S), 2 µl T7 RNA Polymerase Mix (HiScribe T7 Quick High Yield RNA Synthesis Kit, NEB, no. E2050S), and 18 µl UltraPure water. The reaction was incubated at 37°C for 16 hours and then held at 4°C before purification. crRNA was then purified using TRI Reagent (Sigma-Aldrich, no. T9424-25ML) according to the manufacturer's protocol. The quality of crRNA was assessed using RNA ScreenTape for the 4200 TapeStation system (Agilent, no. 5067-5576). The best-performing RPL13a crRNA was determined using SHERLOCK, which was then selected for high-quality synthesis by IDT (Ultramer RNA Oligo, 4 nmol, standard desalting).

For crRNA screening, DFT1, DFT2, RPL13a and ZIKV templates were pre-amplified with RPA primers by PCR. DFT1 and DFT2 targets were amplified from DFT1 and DFT2 cell lines, while the RPL13a target was amplified from a devil primary fibroblast culture. ZIKV template was amplified from a short synthetic DNA fragment (IDT). The resulting target for IVT and CRISPR-Cas13a activation and cleaving (hereafter referred to as the CRISPR reaction) contained a T7 sequence appended at the 5′ end. In total, 10 ng of each template was used in the CRISPR reaction. In addition to the no-template control (NTC), crRNA was mispaired to wrong targets to assess the specificity of the detection. The incubation and fluorescence reading were carried out at 37°C for up to 3 hours using black 384-well flat, clear-bottom plates in a Spark 20M (Tecan) plate reader.

### 2.7. CRISPR reaction mix composition and detection

The SHERLOCK ready mix was prepared in an RNase-free environment. For fluorescent plate viewing, the reaction was carried out in a black 384-well flat, clear-bottom, black plate (Corning). A Spark 20M (Tecan) plate reader was set to a kinetic fluorescence reading mode with 490 nm excitation (bandwidth of 10 nm) and 520 nm emission wavelength (bandwidth of 15 nm) with a manual gain setting of 100 (with gain regulation, 30 flashes and 40 µs integration time). Z-position was set to automatically calculate from well A1. The stage temperature was set to 37°C with a 1-minute measurement interval for 1 hour or a 5-minute measurement interval over 3 hours. Each reaction (20 µl per well) consists of 20 mM HEPES buffer (0.4 µl of 1 M stock, pH adjusted to 6.8, Gibco), 9 mM MgCl_2_ (0.18 µl of 1M stock, Invitrogen), 1 mM ribonucleotide solution mix (0.8 µl of 25 mM stock, NEB, no. N0466S), 6.33 µg ml^−1^ LwCas13a (2 µl of 63.3 µg ml^−1^ stock), 2 units µl^−1^ Murine RNase inhibitor (1 µl of 40 units µl^−1^ stock, NEB, no. M0314L), 1.25 units µl^−1^ T7 RNA polymerase (0.5 µl of 50 units µl^−1^ stock, NEB, no. M0251S), 0.5 ng µl^−1^ crRNA specific to the target of interest (1 µl of 10 ng µl^−1^ stock), 0.125 µM RNA reporter (1.25 µl of 2 µM stock), 1.6 µl post amplification RPA product, and 11.27 µl UltraPure water (Invitrogen). RNaseAlert Lab Test Kit v2 (Thermo Fisher Scientific) and RNaseAlert Substrate (IDT) were used as the RNA reporter. The reporter was reconstituted to 2 µM by adding 2.5 µl of 10X RNaseAlert buffer and 22.5 µl of UltraPure water.

For handheld fluorescence viewing amenable to field conditions, the reaction was carried out in 200-µl PCR tubes and incubated on a heat block or thermal cycler at 37°C for up to 3 hours. Tubes were visualized using P51 Molecular Fluorescence Viewer (minipcrbio) equipped with a blue LED strip and amber filter. Each reaction (20 µl per tube) consists of 20 mM HEPES buffer (0.4 µl of 1 M stock, pH adjusted to 6.8, Gibco), 9 mM MgCl_2_ (0.18 µl of 1M stock, Invitrogen), 1 mM ribonucleotide solution mix (0.8 µl of 25 mM stock, NEB, no. N0466S), 6.33 µg ml^−1^ LwCas13a (2 µl of 63.3 µg ml^−1^ stock), 2 units µl^−1^ Murine RNase inhibitor (1 µl of 40 units µl^−1^ stock, NEB, no. M0314L), 1.25 units µl^−1^ T7 RNA polymerase (0.5 µl of 50 units µl^−1^ stock, NEB, no. M0251S), 0.5 ng µl^−1^ crRNA specific to the target of interest (1 µl of 10 ng µl^−1^ stock), 0.5 µM RNA reporter (5 µl of 2 µM stock), 1.6 µl post amplification RPA product and 7.52 µl UltraPure water (Invitrogen ).

### 2.8. Limit-of-detection

Limit-of-detection (LOD) was determined for SHERLOCK targeting DFT1, DFT2 and RPL13a. For each SHERLOCK assay, gDNA isolated from DFT1, DFT2 and fibroblast cells (for RPL13a) was serially diluted from 1 fM (10^−15^ M) down to 1 zM (10^−21^ M). Copy number was estimated using DNA Copy Number and Dilution Calculator (Thermo Fisher Scientific) based on the estimated Tasmanian devil's genome size of 3 gigabase pairs [32]. A total of 2 µl of the template from each concentration was added into the corresponding RPA reactions and incubated for 30 minutes. RPA products were then added into corresponding SHERLOCK ready mixes before being incubated in the Spark 20M (Tecan) plate reader with a 5-minute reading interval for 3 hours, as previously described.

## 3. Results

### 3.1. Overview of the SHERLOCK diagnostic assay for DFT1 and DFT2

Our SHERLOCK assay consists of three separate tubes. Tube 1 for crude DNA extraction, tube 2 for target DNA amplification and tube 3 for the conversion of DNA to RNA and CRISPR-Cas13a nucleic acid detection (figure 1). For tube 1, primary samples are incubated in S-PREP buffer at 95°C for 10 minutes (figure 1A,B). The crude DNA sample from tube 1 is then added to tube 2 to amplify the target DNA with recombinase polymerase amplification (RPA) at 37°C for 30 minutes (figure 1A). RPA uses paired forward and reverse primers to amplify DFT1- or DFT2-specific markers, alongside primers specific to a housekeeping gene (RPL13a), which serves as a positive control (figure 1A). The 5’ RPA primer also includes a T7 polymerase recognition site sequence, which is needed to convert DNA to RNA during the *in vitro* transcription (IVT) in tube 3 (figure 1A). Tube 3 also contains the LwCas13a enzyme, the CRISPR-RNA (crRNA) specific to DFT1, DFT2, or RPL13, and a quencher linked to a fluorophore by a short RNA strand (RNAseAlert, Integrated DNA Technologies). Recognition of the target sequence by the crRNA activates the LwCas13a enzyme to cleave the RNA linking the quencher to the fluorophore. The conversion of DNA to RNA and detection of nucleic acid occur simultaneously in tube 3 as it is incubated at 37°C from 5 minutes to 3 hours, depending on the concentration of the target DNA (figure 1A). Biopsies are taken from the tumour site or control site (figure 1B shows an oral mucosal swab being performed). The test result is visualized using a handheld fluorescence viewer (minipcrbio) equipped with blue LED lights (figure 1C). All aforementioned steps can be performed in a field setting with limited instrumentation (figure 1D). The key components needed are heat blocks (which can be battery-powered), micropipettes (10 µl), and a handheld P51 Molecular Fluorescence Viewer (MiniPCR Bio) that can be powered by batteries or a USB-C connection.

**Figure 1.**
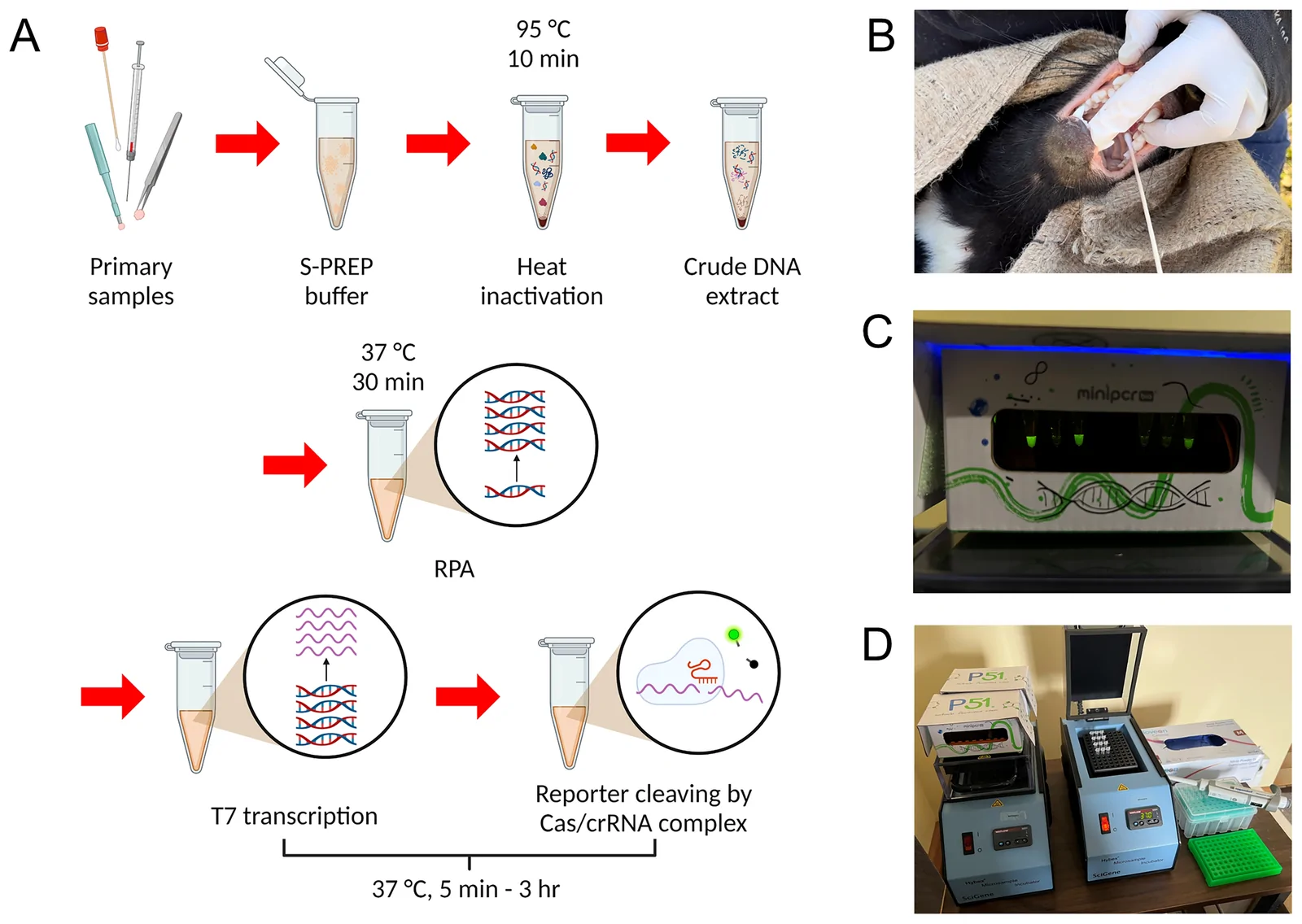
SHERLOCK field testing overview. (A) Schematic of the SHERLOCK assay consisting of sample preparation involving the deactivation of primary samples in S-PREP buffer at 95°C for 10 minutes, DNA amplification using RPA at 37°C for 30 minutes, and T7 transcription and reporter cleaving at 37°C for 5 minutes to 3 hours. (B) An oral mucosa swab sample was taken from a wild Tasmanian devil. (C) SHERLOCK tests visualized using a handheld fluorescence viewer. (D) Equipment needed to perform the field SHERLOCK diagnosis.

### 3.2. Screening of RPA primers, LwCas13a and crRNA

RPA primers were screened to optimize primer pairs using RPA followed by gel electrophoresis. Six, three and four RPA primer pairs for DFT1, DFT2 and RPL13a were selected based on the gel electrophoresis results. These primers were then tested with the sgRNA and LwCas13a in the SHERLOCK reaction, which demonstrated that they efficiently amplified the target of interest, leading to CRISPR nucleic acid detection and reporter cleaving (supplementary material, figures S2–S4).

The purified LwCas13a collateral cleaving ability was first tested by pairing crRNA to corresponding targets amplified by PCR, with a ZIKV DNA fragment as a positive control. LwCas13a functioned as expected, with the fluorescence output rapidly increasing when pairing with ZIKV crRNA in the presence of ZIKV DNA fragment (figure 2A). Additionally, the fluorescence kinetics showed that LwCas13a paired with our DFT1 and DFT2 crRNA led to a robust detection of the target of interest (figure 2A). As the efficiencies of DFT1 and DFT2 crRNA were comparable to the ZIKV crRNA previously developed and validated by Kellner *et al.* [30], we did not perform any further crRNA screening. Mispairing DFT1 and DFT2 crRNAs and targets did not result in activation of the CRISPR reaction, as shown by the lack of fluorescence increase above background (figure 2A). This suggested that each reaction is highly specific to the target of interest.

**Figure 2.**
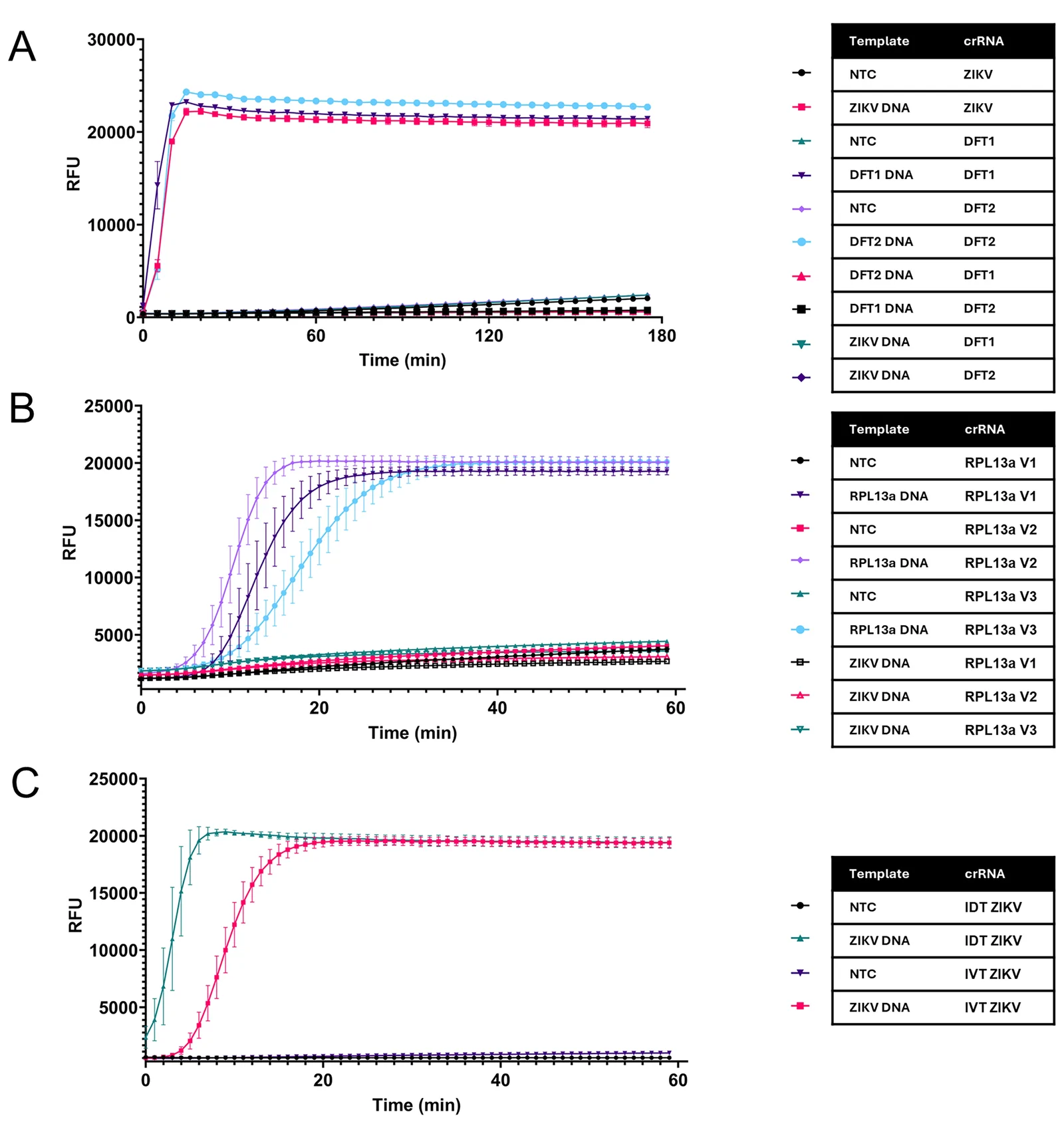
CrRNA specificity for DFT1, DFT2 and RPL13a using purified DNA. (A) DFT1, DFT2 and ZIKV CRISPR reaction. crRNA was paired with the corresponding target, or water as a no-template control (NTC). Additionally, crRNAs were paired with incorrect targets to assess the detection specificity. (B) Screening of *in vitro* transcribed (IVT) RPL13a crRNAs. All IVT crRNAs were paired with their correct target or water (NTC). The specificity of all IVT crRNAs was assessed by mispairing with ZIKV DNA. (C) Assessing the efficiency between IVT crRNA and IDT-synthesized crRNA. The crRNAs were either paired with their corresponding target or water (NTC). All reactions were assessed according to fluorescence kinetics of relative fluorescence units (RFU) over time. *n* = 2, error bar = standard deviation.

For RPL13a, we designed 3 crRNA DNA templates to be *in vitro* transcribed. The quality of all IVT crRNAs was assessed using RNA ScreenTape for the 4200 TapeStation system (Agilent). The results showed that all IVT reactions produced a sharp single RNA band with the correct size (supplementary material, figure S5). All three IVT RPL13a crRNAs produced detectable fluorescent signals when paired with their corresponding target, with the RPL13a V2 crRNA being the most efficient crRNA (figure 2B). The specificity was again assessed by mispairing all crRNAs to the wrong target (ZIKV DNA in this case). The result suggested that all RPL13a crRNAs were highly specific for their target, as indicated by the lack of fluorescence increase above background (figure 2B). It was noted that the background fluorescence signal relative to the positive signal was higher for IVT crRNAs than for commercially purchased crRNAs (figure 2B). The efficiency between IVT crRNA and custom-ordered crRNA (IDT) was additionally assessed. The commercially purchased crRNA had a higher detection efficiency than IVT crRNA despite the latter being of high quality and purity (figure 2C). Therefore, we decided to custom order the RPL13a V2 crRNA to be able to achieve the highest sensitivity for our SHERLOCK tests.

### 3.3. The effects of S-PREP buffer on downstream SHERLOCK detection

To assess the need for heating the samples in S-PREP buffer, the plasma samples from a healthy devil were spiked with DFT1 genomic DNA or DFT2 genomic DNA to simulate primary samples collected from DFT1- or DFT2-affected animals. The results revealed that SHERLOCK was able to detect its targets even without sample inactivation with S-PREP buffer at 95°C (figure 3). However, the rate of detection was markedly slower compared to S-PREP-inactivated buffer (figure 3). DFT1 and DFT2 SHERLOCK reached the maximum fluorescence signal after 180 and 100 minutes of incubation, respectively, without S-PREP inactivation (figure 3A,B). The maximum fluorescence signal was achieved within 30 minutes of incubation with S-PREP inactivation (figure 3A,B).

**Figure 3.**
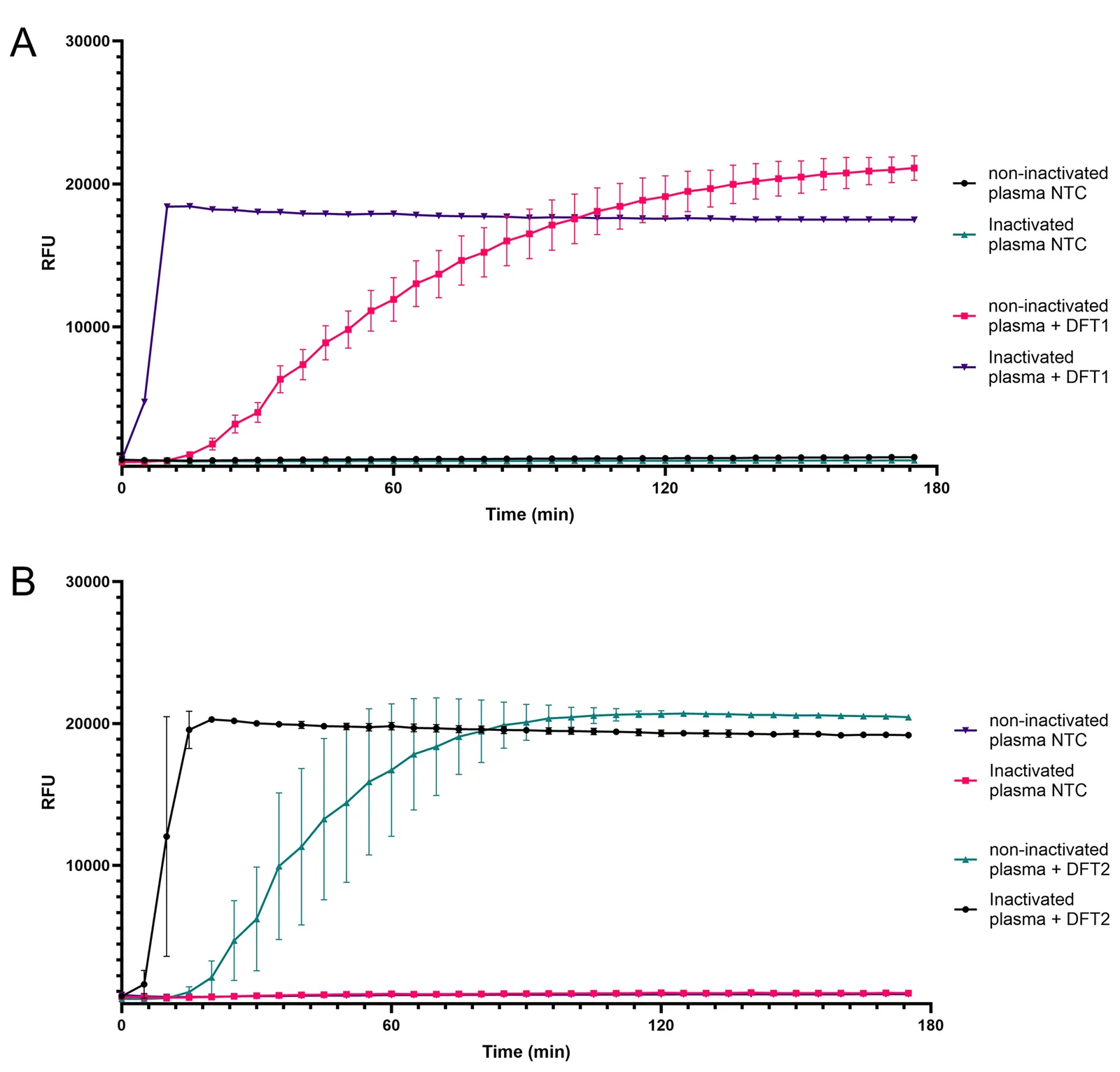
S-PREP versus no S-PREP for sample extraction and inactivation. Fluorescence kinetics (relative fluorescence units, RFU) over time. (A) Non-inactivated versus S-PREP-inactivated plasma samples with or without spiked-in DFT1 genomic DNA. (B) Non-inactivated versus S-PREP inactivated plasma samples with or without spiked-in DFT2 genomic DNA. NTC denotes no template control. *n* = 2, error bar = standard deviation.

### 3.4. SHERLOCK testing of DFT and non-DFT tumour samples

The ability to distinguish DFT from non-DFT tumours is vital to monitor the spread of DFT1 and DFT2. We included two non-DFT tumour biopsies, an anal sac tumour and a round cell tumour, alongside ear biopsies from healthy devils, and performed a set of SHERLOCK tests following S-PREP inactivation and extraction. Results revealed that only when SHERLOCK was paired with the correct target, DFT1 or DFT2, it would lead to a positive detection indicated by the fluorescence increase (figure 4). These findings are consistent with the analytical specificity of the SHERLOCK assays for the limited samples evaluated, although additional testing with more non-DFT tumours will be needed to further validate assay specificity (figure 4).

**Figure 4.**
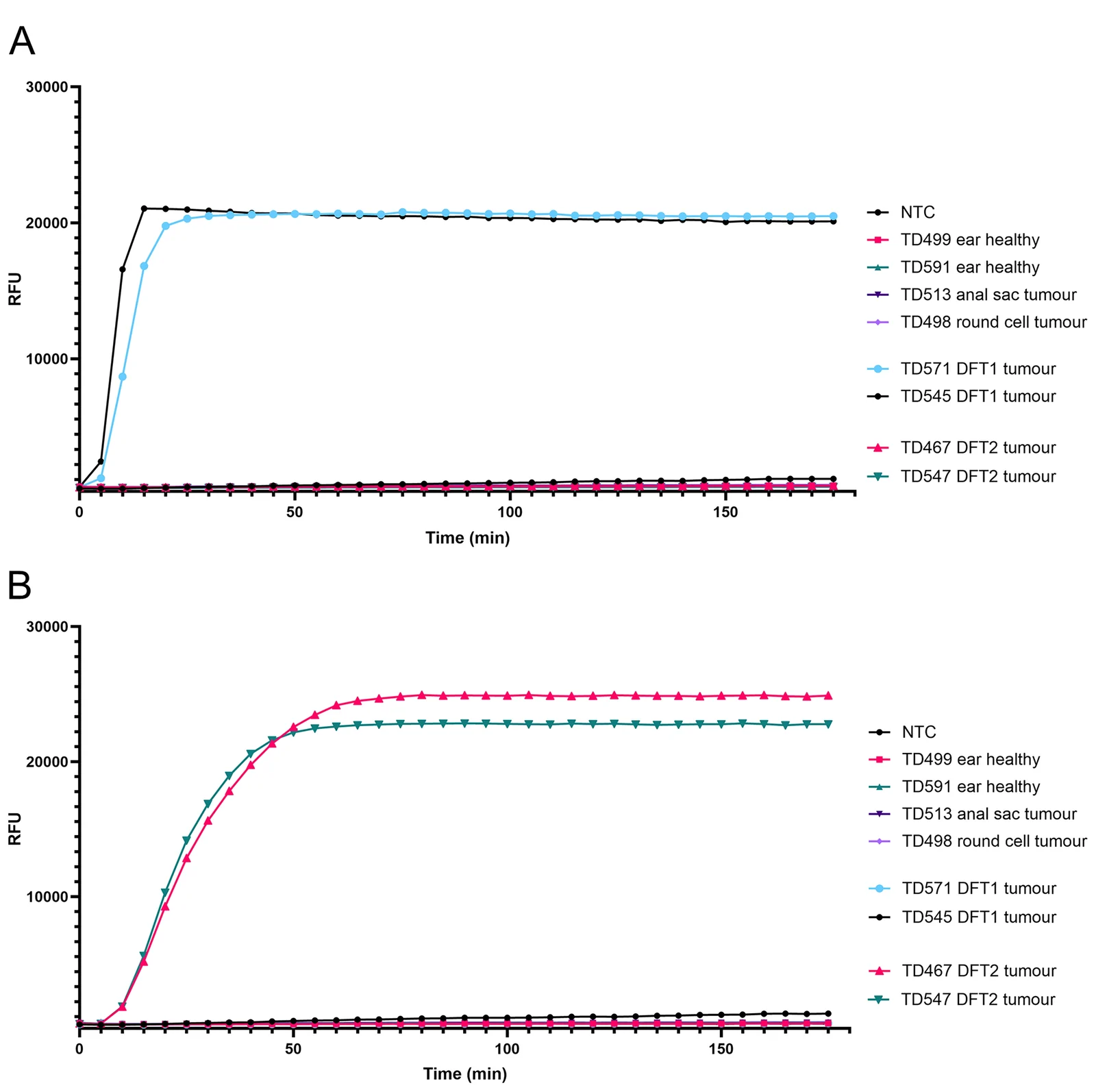
SHERLOCK on DFT1/2, non-DFT tumours and healthy tissue biopsies. (A) DFT1 SHERLOCK tested on DFT1 and DFT2, anal sac tumour, round cell tumour and healthy ear biopsies. (B) DFT2 SHERLOCK tested on DFT1 and DFT2, anal sac tumour, round cell tumour and healthy ear biopsies. NTC and TD denote no template control and Tasmanian devil ID, respectively. Reactions were assessed as fluorescence kinetics of relative fluorescence units (RFU) over time. *n* = 1.

### 3.5. SHERLOCK is highly sensitive

The sensitivity of SHERLOCK assays for DFT1, DFT2 and RPL13a was determined using serially diluted gDNA extracted from DFT1, DFT2 and fibroblast cells. We found that all three SHERLOCK assays could detect their respective target as low as 10 attomolar (aM) concentration, which is approximately the equivalent of six copies of DNA in a 20 µl SHERLOCK reaction (figure 5). No fluorescent signal higher than the background was detected in reactions at or below 1 aM within 180 minutes of reaction time (figure 5).

**Figure 5.**
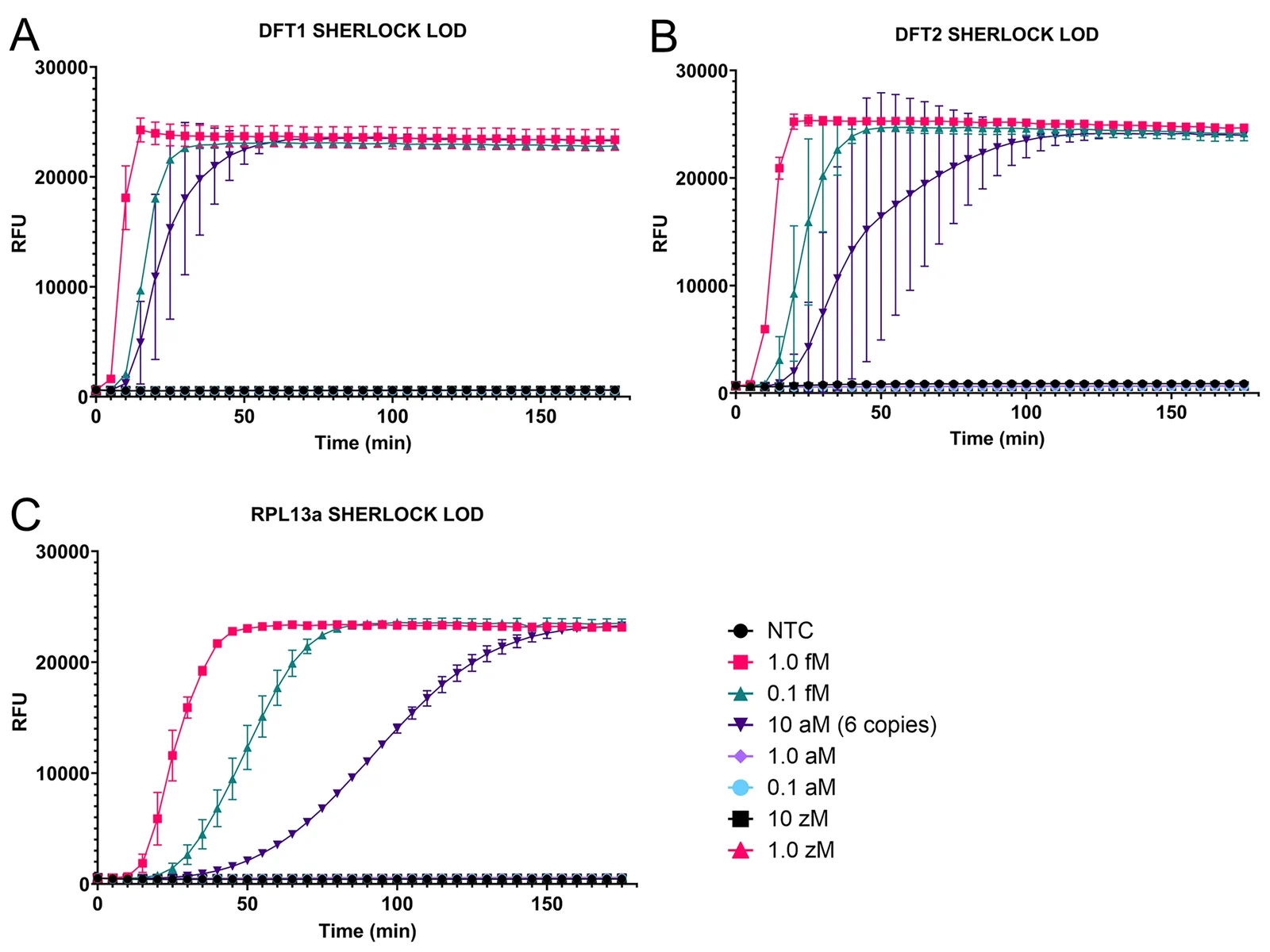
Limit-of-detection (LOD) of SHERLOCK for DFT1, DFT2 and RPL13a. Fluorescence kinetics (relative fluorescence units; RFU) over time (minutes; min) of three SHERLOCK assays. A serially diluted gDNA from DFT1, DFT2 and fibroblast cell lines from 1 femtomolar (fM) down to 1 zeptomolar (zM) was used to determine the LOD. (A) SHERLOCK for DFT1, (C) SHERLOCK for DFT2 and (B) SHERLOCK for RPL13a. NTC denotes no template control. *n* = 2, error bar = standard deviation.

### 3.6. Troubleshooting and validation of field SHERLOCK testing on post-mortem samples

We validated our SHERLOCK DFT1 and DFT2 tests on post-mortem samples from deceased devils. Note that this was performed without the RPL13a control, which was not yet available at this time point. We performed SHERLOCK on biopsy and swab samples. Varying sizes of tumour biopsies were collected from six devils with suspected DFT1 and DFT2 masses before being added to microcentrifuge tubes with S-PREP buffer (figure 6A). SHERLOCK tests with a biopsy of 5 mm^3^ or larger yielded a negative result, whereas a biopsy smaller than 3 mm^3^ led to a positive detection of DFT1 and DFT2 in the first testing (figure 6A). Samples from devils TD618, TD619 and TD622 (5 mm^3^ biopsies) were retested by diluting the crude lysate sample solution from the first test into a 1:1 dilution in fresh S-PREP buffer. All three samples then tested positive for DFT1 (figure 6A), in agreement with the PCR testing on extracted DNA. This suggests that inhibitory substances in the crude lysate may have inhibited the SHERLOCK reaction.

**Figure 6.**
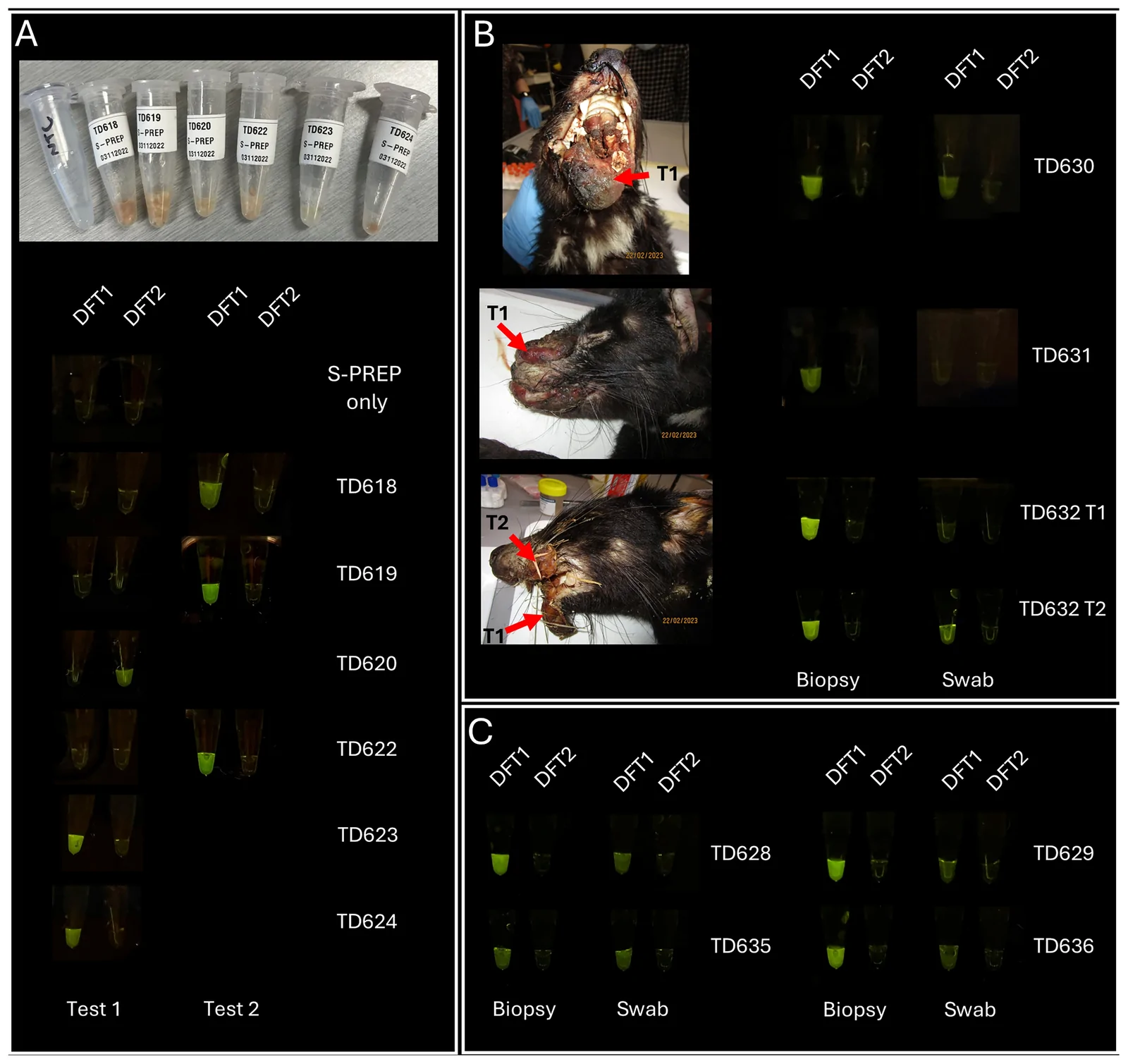
SHERLOCK testing on post-mortem samples. A positive detection for DFT1 or DFT2 is indicated by the visible green fluorescence. TD denotes Tamanian devil identification numbers. (A) Tumour biopsy samples from six devils in S-PREP buffer and the corresponding SHERLOCK results. ‘S-PREP only’ indicates SHERLOCK on a no-template control (NTC). Samples from TD618, TD619 and TD622 demonstrated a false-negative result that was eliminated when the sample was diluted 1:1 and re-tested. (B) Pictures of three devils and corresponding SHERLOCK results from tumour biopsies and swab samples. Red arrows indicate where tumour samples were collected (T1 and T2 denote tumour 1 and tumour 2, respectively). (C) SHERLOCK on biopsy and swab samples from four devils. All biopsy and swab samples tested positive for DFT1.

Four swab and biopsy samples were taken from three deceased devils to assess the viability of SHERLOCK on swab samples (figure 6B). Swab samples were taken from the area indicated on figure 6B without cleaning the area prior to sample collection. Only two of four swab samples tested positive for DFT1, while all biopsy samples, the size of 1 mm^3^, produced a DFT1-positive detection (figure 6B). Sample quality, apart from the concentration of the DNA, is an important consideration. We hypothesized that lower-quality samples, especially necrotic and/or with secondary microbial infection, may contain inhibitory compounds that can cause false negative results.

Lastly, we tested our SHERLOCK on four additional deceased animals to validate the revised sampling procedures. Specifically, swab samples were collected from lesions with less visible necrosis and microbial contamination, and 1 mm^3^ biopsies were collected to minimize reaction inhibition and to confirm swab test results. Figure 6C shows that DFT1 was detected in all swab and biopsy samples for devils TD623, TD624, TD628 and TD635. The swabs tested positive but with lower fluorescence intensity than the biopsy samples at the time points used in this experiment (figure 6C). Table 1 summarizes SHERLOCK test results from post-mortem samples.

**Table 1.** Result summary for SHERLOCK testing on post-mortem tumour samples.

	devil ID	clinical presentation	PCR diagnosis	sample type	DFT1 detection	DFT2 detection
figure 6A	TD618	DFTD	DFT1	biopsy	+^*^	−
	TD619	DFTD	DFT1	biopsy	+^*^	−
	TD620	DFTD	DFT2	biopsy	−	+
	TD622	DFTD	DFT1	biopsy	+^*^	−
	TD623	DFTD	DFT1	biopsy	+	−
	TD624	DFTD	DFT1	biopsy	+	−
figure 6B	TD630	DFTD	DFT1	biopsy	+	−
	TD630	DFTD	DFT1	swab	+	−
	TD631	DFTD	DFT1	biopsy	+	−
	TD631	DFTD	DFT1	swab	−	−
	TD632 T1	DFTD	DFT1	biopsy	+	−
	TD632 T1	DFTD	DFT1	swab	−	−
	TD632 T2	DFTD	DFT1	biopsy	+	−
	TD632 T2	DFTD	DFT1	swab	+	−
figure 6C	TD628	DFTD	DFT1	biopsy	+	−
	TD628	DFTD	DFT1	swab	+	−
	TD629	DFTD	DFT1	biopsy	+	−
	TD629	DFTD	DFT1	swab	+	−
	TD635	DFTD	DFT1	biopsy	+	−
	TD635	DFTD	DFT1	swab	+	−

*Notes*: *, positive detection achieved after the sample was diluted in fresh S-PREP buffer (1:1 dilution); T1 = tumour 1; T2 = tumour 2; + = positive result; − = negative result.

### 3.7. Point-of-care SHERLOCK test on wild animals in the field

We deployed the optimized DFT1, DFT2 and RPL13a SHERLOCK tests to assess performance in the field setting with minimally invasive swab samples and limited instrumentation. However, to demonstrate the versatility of the test, we also included biopsy and fine needle aspirate (FNA) samples. A total of 33 SHERLOCK tests were conducted on 10 devils and three quolls. Figure 7A shows the complete SHERLOCK for DFT1, DFT2 and RPL13A positive control on wild devils with no clinical signs of DFTD (*n* = 3). The samples were positive for RPL13A, but no fluorescence signals were detected for DFT1 or DFT2 SHERLOCK, indicating that they were free of DFTD (figure 7A). Figure 7B shows SHERLOCK with a positive detection of devils affected by DFTD (*n* = 5). The test was able to detect DFT1 infection in all devils with suspected DFT1 masses. However, two samples had to be re-tested owing to a weak positive detection (Nacho) and a weak positive control detection in the FNA sample (Salsa; figure 7B). It was found that the weak fluorescence signal was probably owing to the presence of inhibitors in the reaction. A swab sample from ‘Nacho’ was diluted in fresh S-PREP buffer (1 : 3 dilution) and retested, which revealed a stronger fluorescence intensity (figure 7B). We added approximately six drops of FNA from Salsa into an S-PREP buffer, and it became apparent that the reaction was inhibited, as indicated by a weak fluorescence signal. In a similar manner, an FNA sample from Salsa was diluted in fresh buffer (1 : 1 dilution), which also yielded a strong positive detection for DFT1 (figure 7B).

**Figure 7.**
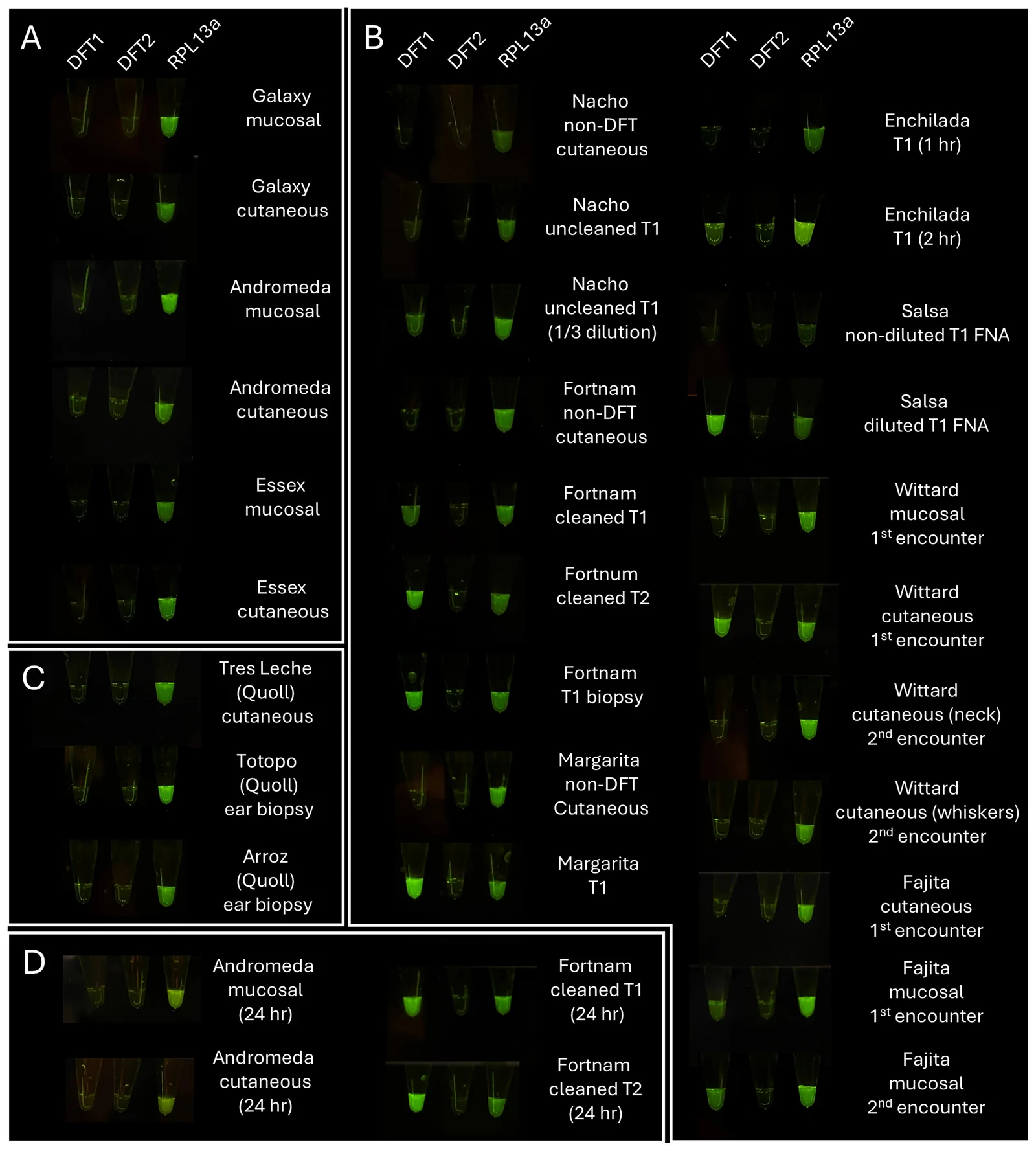
Field SHERLOCK testing on wild animals. Samples from cutaneous (cheek) and oral mucosal swabs were used unless stated otherwise. Positive detections were indicated by the visible green fluorescence. RPL13a served as a positive control. (A) SHERLOCK results from DFT1 and DFT2 negative devils. (B) SHERLOCK results from devils with DFT1. (C) SHERLOCK results on spotted-tail quolls. All samples tested negative for DFT2 at this field site, where DFT2 is not present. (D) SHERLOCK results after 24 hours of incubation. *T1,* tumour number 1*; T2,* tumour number 2*; uncleaned*, sample taken from an area without cleaning with saline and gauze; *cleaned*, sample taken from an area that has been cleaned with saline and gauze; *first encounter*, sample taken from an animal on the first encounter within the same trapping trip; *second encounter*, sample taken from the same animal on a second encounter; *FNA*, fine needle aspirate.

Two devils who appeared healthy and unaffected by DFTD, with no tumours or lesions, tested positive for DFT1. A cutaneous swab sample from Wittard, a 4-year-old male devil, tested positive for DFT1 (figure 7B). We repeated the test from samples taken on the same day to rule out cross-contamination during sample preparation, which also resulted in DFT1 positive (data not shown). We collected more cutaneous samples from the same devil on the second trap encounter 6 days later, which revealed a negative result for DFT1 and DFT2 on both swab samples (figure 7B). Another devil, Fajita, a 1-year-old female devil with four pouch young that appeared healthy, tested positive for DFT1 from the mucosal swab (figure 7B). Fajita was trapped 6 days after the initial encounter, and the mucosal swab sample again returned a DFT1-positive result (figure 7B). Upon a closer inspection of the oral cavity, we identified a small non-ulcerated discoloured mark along the gum line (supplementary material, figure S6). However, this did not appear to have the clinical presentation resembling DFT1 or DFT2 lesion. All swabs taken from the lateral region away from any visible tumour (or area where DFT1 was detected in cases with no clinical sign of DFTD) returned negative results for DFT1 and DFT2 (*n* = 5). Additionally, the field was more than 200 km from regions known to have DFT2, and as expected, all 33 samples tested negative for DFT2 at this site.

We conducted SHERLOCK on swabs and biopsies taken from spotted-tail quolls (*Dasyurus maculatus*) to assess whether RPL13a can be detected and to help assess the false positive rate for the DFT1 and DFT2 tests. All swabs and biopsies tested positive for RPL13a, including biopsies taken from spotted-tail quolls, suggesting that the RPL13a positive control test can work for multiple *Dasyuridae* species (figure 7C).

To test whether the fluorescence signal was observable after an extended period of incubation, DFT1 negative and positive samples from two devils were incubated for 24 hours. Figure 7D shows that the fluorescence signal remained visible after 24 hours, and no positive detection was observed for reactions that were initially negative. These results suggest that extended incubation did not result in false-positive detection under the conditions and samples tested. However, further validation using a larger number of samples would be required to confirm the impact of prolonged incubation on assay specificity and fluorescence reporter stability.

Based on these field-testing results, optimal assay performance was achieved by limiting solid and liquid biopsy inputs to approximately 1 mm³ and 100 µl, respectively, to minimize reaction inhibition. Although extending the incubation time to 24 hours did not result in false-positive detection among the samples evaluated, an incubation period of 3 hours was sufficient for reliable interpretation of results. Table 2 summarizes SHERLOCK field test results from live animals.

**Table 2.** Result summary for field SHERLOCK testing on wild animals.

	devil ID	species	clinical presentation	sample type	tissue type	incubation time (hour)	DFT1 detection 1st encounter	DFT2 detection 1st encounter	RPL13a detection 1st encounter	DFT1 detection 2nd encounter	DFT2 detection 2nd encounter	RPL13a detection 2nd encounter
figure 7A	Galaxy	*Sarcophilus harrisii*	non-DFTD	swab	non-DFTD mucosal	1	−	−	+	NA	NA	NA
	Galaxy	*Sarcophilus harrisii*	non-DFTD	swab	non-DFTD cutaneous	1	−	−	+	NA	NA	NA
	Andromeda	*Sarcophilus harrisii*	non-DFTD	swab	non-DFTD mucosal	1	−	−	+	NA	NA	NA
	Andromeda	*Sarcophilus harrisii*	non-DFTD	swab	non-DFTD cutaneous	1	−	−	+	NA	NA	NA
	Essex	*Sarcophilus harrisii*	non-DFTD	swab	non-DFTD mucosal	1	−	−	+	NA	NA	NA
	Essex	*Sarcophilus harrisii*	non-DFTD	swab	non-DFTD cutaneous	1	−	−	+	NA	NA	NA
figure 7B	Nacho	*Sarcophilus harrisii*	DFTD	swab	non-DFTD cutaneous	1	−	−	+	NA	NA	NA
	Nacho	*Sarcophilus harrisii*	DFTD	swab	uncleaned T1	1	+^*^	−	+	NA	NA	NA
	Nacho	*Sarcophilus harrisii*	DFTD	swab	uncleaned T1 (1/3 dilution)	1	+	−	+	NA	NA	NA
	Fortnam	*Sarcophilus harrisii*	DFTD	swab	non-DFTD cutaneous	1	−	−	+	NA	NA	NA
	Fortnam	*Sarcophilus harrisii*	DFTD	swab	cleaned T1	1	+	−	+	NA	NA	NA
	Fortnam	*Sarcophilus harrisii*	DFTD	swab	cleaned T2	1	+	−	+	NA	NA	NA
	Fortnam	*Sarcophilus harrisii*	DFTD	biopsy	T1	1	+	−	+	NA	NA	NA
	Margarita	*Sarcophilus harrisii*	DFTD	swab	non-DFTD cutaneous	1	−	−	+	NA	NA	NA
	Margarita	*Sarcophilus harrisii*	DFTD	swab	T1	1	+	−	+	NA	NA	NA
	Enchilada	*Sarcophilus harrisii*	DFTD	swab	T1	1	−	−	+	NA	NA	NA
	Enchilada	*Sarcophilus harrisii*	DFTD	swab	T1	2	+	−	+	NA	NA	NA
	Salsa	*Sarcophilus harrisii*	DFTD	fna	T1	1	−	−	+^*^	NA	NA	NA
	Salsa	*Sarcophilus harrisii*	DFTD	fna	T1 (1/1 dilution)	1	+	−	+	NA	NA	NA
	Wittard	*Sarcophilus harrisii*	non-DFTD	swab	non-DFTD mucosal	1	−	−	+	NA	NA	NA
	Wittard	*Sarcophilus harrisii*	non-DFTD	swab	non-DFTD cutaneous	1	+	−	+	NA	NA	NA
	Wittard	*Sarcophilus harrisii*	non-DFTD	swab	non-DFTD cutaneous (neck)	1	NA	NA	NA	−	−	+
	Wittard	*Sarcophilus harrisii*	non-DFTD	swab	non-DFTD cutaneous (whiskers)	1	NA	NA	NA	−	−	+
	Fajita	*Sarcophilus harrisii*	non-DFTD	swab	non-DFTD cutaneous	1	−	−	+	NA	NA	NA
	Fajita	*Sarcophilus harrisii*	non-DFTD	swab	non-DFTD mucosal	1	+	−	+	NA	NA	NA
	Fajita	*Sarcophilus harrisii*	non-DFTD	swab	non-DFTD mucosal	1	NA	NA	NA	+	-	+
figure 7C	Tres Leche	*Dasyurus maculatus*	non-DFTD	swab	non-DFTD cutaneous	1	+	−	+	NA	NA	NA
	Totopo	*Dasyurus maculatus*	non-DFTD	biopsy	non-DFTD ear	1	+	−	+	NA	NA	NA
	Arroz	*Dasyurus maculatus*	non-DFTD	biopsy	non-DFTD ear	1	+	−	+	NA	NA	NA
figure 7D	Andromeda	*Sarcophilus harrisii*	non-DFTD	swab	non-DFTD mucosal	24	−	−	+	NA	NA	NA
	Andromeda	*Sarcophilus harrisii*	non-DFTD	swab	non-DFTD cutaneous	24	−	−	+	NA	NA	NA
	Fortnam	*Sarcophilus harrisii*	DFTD	swab	cleaned T1	24	+	−	+	NA	NA	NA
	Fortnam	*Sarcophilus harrisii*	DFTD	swab	cleaned T2	24	+	−	+	NA	NA	NA

*Notes*: *, low positive detection; T1, tumour 1; T2, tumour 2; FNA, fine needle aspirate; +, positive result; −, negative result; NA, not applicable.

## 4. Discussion

The SHERLOCK tests for DFT1, DFT2 and RPL13a yielded reliable results in the field and laboratory using a diverse set of input samples, including purified DNA, cell lines, biopsies and swabs. The potential to diagnose animals in the field with minimal instrumentation could be useful for understanding latent infections and making near real-time conservation management decisions. However, many additional samples will need to be collected, and the results of other testing methods, such as PCR, immunohistochemistry and sequencing-based approaches, will need to be directly compared to fully validate SHERLOCK as a diagnostic test.

As DFT1 is present at all field sites and infections can be latent for several months, identifying true positive and negative DFT1 samples is challenging. However, DFT2 is only present in southern Tasmania. Encouragingly, no positive DFT2 detections were observed across 33 field SHERLOCK tests performed at sites in northern Tasmania where DFT2 is not present, suggesting a low false positive rate for DFT2. Future studies could assess true false positive rates for DFT1 and DFT2 by using samples from the wild devil insurance population on Maria Island off the east coast of Tasmania, where neither DFT1 nor DFT2 are present. Additionally, the RPL13a results were consistently positive, suggesting high true positive and low false positive rates for this critical control test. Additionally, laboratory validation using purified DNA showed that the assays could detect as few as six copies of DNA per 20 µl reaction, demonstrating high analytical sensitivity. This is on par with the SHERLOCK test for SARS-CoV-2 kit that gained the emergency use authorization from the FDA (LOD of 6.75 copies of genomic viral RNA) [33].

Two wild devils, Wittard and Fajita, that appeared to be healthy tested positive for DFT1. Both Wittard and Fajita were re-trapped 6 days after the initial positive DFT1 test. Fajita again tested positive for DFT1, and upon further inspection, a small non-ulcerated discolouration was detected in the oral cavity. Although the appearance of the discolouration did not resemble a typical DFT1 lesion, there is currently limited data on the early visual indicators of DFT1, partly owing to the lack of rapid point-of-care tests. By contrast, Wittard tested negative upon re-sampling, and the initial positive result cannot be explained with the data currently available. The positive results from these devils should therefore be interpreted with caution, but open the door to detection of potential latent infections or recent exposures to transmissible cancer cells.

Several explanations are possible, including an early stage of the disease that was not clinically recognizable, contamination, or false positive detection. For Wittard, a devil with severe DFT1 (Nacho) was trapped within 100 metres, making it possible that DFT1 DNA was transferred via direct contact or from residual environmental DNA. However, this explanation remains speculative. Similarly, while repeated DFT1 positive detection in Fajita and the absence of DFT1 positive signal from a region away from the discoloured mark provide some support for an early DFT1 detection, the current sample size is not sufficient to distinguish between these possibilities. Future studies incorporating longitudinal monitoring and confirmation using established diagnostic methods, such as Tasman PCR, will be required to determine whether positive SHERLOCK results in animals without visible tumours represent genuine pre-clinical detection or other sources of positive signal. If confirmed, SHERLOCK may have potential as a tool for detecting DFTD prior to the development of overt clinical signs, which could improve disease surveillance and conservation management.

The minimal instrumentation required for SHERLOCK makes this the most feasible field diagnostic test to date. Additionally, the protocol is relatively simple compared to other techniques such as PCR. Thus, SHERLOCK tests for other pathogens could be used for other wildlife studies and in veterinary facilities that lack molecular biology equipment. Additional studies are needed to compare the sensitivity and specificity of alternative diagnostic methods. We found that sample extraction and inactivation with S-PREP is needed to extract crude gDNA and to prevent the inhibition of RPA and downstream CRISPR detection. This was observed when comparing S-PREP inactivated and non-inactivated plasma samples spiked with DFT1 or DFT2 gDNA. Additionally, a study led by Lee *et al.* [28] reported that non-inactivated samples could also result in a false positive detection. This was the case with samples with high levels of nucleases, causing nonspecific cleavage of the RNA reporter. As a rapid test is highly desirable, future studies can examine the minimum time needed for incubation and heating in S-PREP buffer.

Our field testing demonstrated the versatility of SHERLOCK, which is compatible with various types of samples such as solid/liquid biopsy and swab samples. For primary biopsies, it was evident that too much starting sample could lead to inhibition of SHERLOCK, likely owing to the excess inhibitory molecules. This was eliminated when less sample was used or when the inactivated sample was diluted with fresh S-PREP buffer. We discovered that swab and FNA samples with a secondary infection are prone to giving a false negative result. Therefore, samples should be taken from areas with less necrosis whenever possible.

There are currently no management options to stop DFT2 from spreading across Tasmania, as DFT1 has done in the past 30 years. Samples from deceased animals in the DFT2 zone suggested that the SHERLOCK test can detect DFT2 in the simulated field settings. Pending further validation, the ability to detect DFT2 in the field could pave the way towards active management approaches that are not currently possible. For example, several therapeutic and vaccine approaches have been tested or are in development, and a devil that tested positive in the field for early-stage DFT2 could be administered a vaccine or therapy in the field [34–37].

Future studies should directly compare the specificity and sensitivity of the Tasman PCR test and SHERLOCK test for each sample collected. In the first case, the two tests could be run using aliquots from the same swab sample. Other diagnostic methods such as Oxford Nanopore sequencing may be used as an alternative to SHERLOCK or to validate SHERLOCK findings. However, the technique requires multistep preparation compared to SHERLOCK. Previous studies have shown that mass spectrometry could be used to identify DFT1- and DFT2-associated proteins in serum samples, and has the potential to detect pre-clinical DFTD [38,39].

SHERLOCK testing on animals without clinical signs of DFTD, followed by subsequent trapping trips, will be a key validation step for the crucial pre-clinical test. If a devil without visible DFTD symptoms tests positive on SHERLOCK and later develops a facial tumour, this could indicate successful pre-clinical detection. Future studies should aim to establish the latent period of DFTD, provided positive results from asymptomatic animals prove reliable. Disease monitoring is essential for future vaccination trials that inject healthy devils with tumour cells to assess vaccine efficacy. This known infection date will provide an opportunity to more precisely document the latency period and further validate the SHERLOCK tests.

Although the goal of this study was to assess the performance of SHERLOCK testing, future work needs to explore the long-term scalability and real-world implementation. “One-pot” SHERLOCK assays could be developed to simplify the testing procedure and to reduce the risk of cross-contamination associated with opening and closing multiple tubes. This would also make the test more amenable to non-laboratory scientists. Furthermore, a smartphone application can be developed to be able to quantify the fluorescence signal from a handheld fluorescence viewer. To extend shelf life, they can potentially be lyophilized which would allow the test to be stored at room temperature. Lyophilization of the reagents can also potentially simplify the test protocol and reduce the time required to prepare reagents for field testing. Various studies showed that lyophilization is possible, and the same level of sensitivity is retained [28,40,41].

## Supplementary Material



## Data Availability

All data are either found in the paper or uploaded as supplementary material [42].

## References

[1] Hawkins CE *et al.* 2006 Emerging disease and population decline of an island endemic, the Tasmanian devil *Sarcophilus harrisii*. Biol. Conserv. 131, 307–324. (doi:10.1016/j.biocon.2006.04.010)

[2] Brüniche–Olsen A , Jones ME, Burridge CP, Murchison EP, Holland BR, Austin JJ. 2018 Ancient DNA tracks the mainland extinction and island survival of the Tasmanian devil. J. Biogeogr. 45, 963–976. (doi:10.1111/jbi.13214)

[3] White LC , Saltré F, Bradshaw CJA, Austin JJ. 2018 High-quality fossil dates support a synchronous, Late Holocene extinction of devils and thylacines in mainland Australia. Biol. Lett. 14, 20 170 642. (doi:10.1098/rsbl.2017.0642) PMC5803592 29343562

[4] Jones ME , Barmuta LA. 2000 Niche differentiation among sympatric Australian Dasyurid carnivores. J. Mammal. 81, 434–447. (doi:10.1644/1545-1542(2000)081<0434:NDASAD>2.0.CO;2)

[5] Guiler E . 1978 Observations on the Tasmanian Devil, *Sarcophilus harrisi* (Dasyuridae: fvlarsupiala) at Granville Harbour, 1966-75. Pap. Proc. R. Soc. Tasmania 112, 161–188. (doi:10.26749/rstpp.112.161)

[6] Comte S , Carver S, Hamede R, Jones M. 2020 Changes in spatial organization following an acute epizootic: Tasmanian devils and their transmissible cancer. Glob. Ecol. Conserv. 22, e00993. (doi:10.1016/j.gecco.2020.e00993) PMC8218997 34164571

[7] Lazenby BT *et al.* 2018 Density trends and demographic signals uncover the long-term impact of transmissible cancer in Tasmanian devils. J. Appl. Ecol. 55, 1368–1379. (doi:10.1111/1365-2664.13088) PMC6078421 30089931

[8] Pye RJ *et al.* 2016 A second transmissible cancer in Tasmanian devils. Proc. Natl Acad. Sci. USA 113, 374–379. (doi:10.1073/pnas.1519691113) PMC4720317 26711993

[9] Stammnitz MR *et al.* 2018 The origins and vulnerabilities of two transmissible cancers in Tasmanian devils. Cancer Cell 33, 607–619.e15. (doi:10.1016/j.ccell.2018.03.013) PMC5896245 29634948

[10] Kwon YM *et al.* 2018 Tasman-PCR: a genetic diagnostic assay for Tasmanian devil facial tumour diseases. R. Soc. Open Sci. 5, 180 870. (doi:10.1098/rsos.180870) PMC6227955 30473836

[11] Margres MJ *et al.* 2020 Spontaneous tumor regression in Tasmanian devils associated with *RASL11A* activation. Genetics 215, 1143–1152. (doi:10.1534/genetics.120.303428) PMC7404226 32554701

[12] Pye R *et al.* 2016 Demonstration of immune responses against devil facial tumour disease in wild Tasmanian devils. Biol. Lett. 12, 20 160 553. (doi:10.1098/rsbl.2016.0553) PMC5095191 28120799

[13] Wright B , Willet CE, Hamede R, Jones M, Belov K, Wade CM. 2017 Variants in the host genome may inhibit tumour growth in devil facial tumours: evidence from genome-wide association. Sci. Rep. 7, 423. (doi:10.1038/s41598-017-00439-7) PMC5428454 28341828

[14] Kaminski MM , Abudayyeh OO, Gootenberg JS, Zhang F, Collins JJ. 2021 CRISPR-based diagnostics. Nat. Biomed. Engineer. 5, 643–656. (doi:10.1038/s41551-021-00760-7) 34272525

[15] Notomi T . 2000 Loop-mediated isothermal amplification of DNA. Nucleic Acids. Res. 28, 63e–63. (doi:10.1093/nar/28.12.e63) PMC102748 10871386

[16] Piepenburg O , Williams CH, Stemple DL, Armes NA. 2006 DNA detection using recombination proteins. PLoS Biol. 4, e204. (doi:10.1371/journal.pbio.0040204) PMC1475771 16756388

[17] Yan S , Li C, Lan H, Pan D, Wu Y. 2024 Comparison of four isothermal amplification techniques: LAMP, SEA, CPA, and RPA for the identification of chicken adulteration. Food Control 159, 110302. (doi:10.1016/j.foodcont.2024.110302)

[18] Pang J , Wang Q, Fei Y, Zhu P, Qiao L, Huang H, Dang C, Gao W. 2019 A real-time recombinase polymerase amplification assay for the rapid detection of *Vibrio harveyi*. Mol. Cell. Probes 44, 8–13. (doi:10.1016/j.mcp.2019.01.001) 30610902

[19] Cabada MM , Malaga JL, Castellanos-Gonzalez A, Bagwell KA, Naeger PA, Rogers HK, Maharsi S, Mbaka M, White AC. 2017 Recombinase polymerase amplification compared to real-time polymerase chain reaction test for the detection of *Fasciola hepatica* in Human stool. Am. J. Trop. Med. Hyg. 96, 341–346. (doi:10.4269/ajtmh.16-0601) PMC5303034 27821691

[20] Zou Y , Mason MG, Botella JR. 2020 Evaluation and improvement of isothermal amplification methods for point-of-need plant disease diagnostics. PLoS ONE 15, e0235216. (doi:10.1371/journal.pone.0235216) PMC7323990 32598374

[21] Mojica FJM , Díez-Villaseñor C, García-Martínez J, Soria E. 2005 Intervening sequences of regularly spaced prokaryotic repeats derive from foreign genetic elements. J. Mol. Evol. 60, 174–182. (doi:10.1007/s00239-004-0046-3) 15791728

[22] Pourcel C , Salvignol G, Vergnaud G. 2005 CRISPR elements in *Yersinia pestis* acquire new repeats by preferential uptake of bacteriophage DNA, and provide additional tools for evolutionary studies. Microbiology 151, 653–663. (doi:10.1099/mic.0.27437-0) 15758212

[23] Mali P , Yang L, Esvelt KM, Aach J, Guell M, DiCarlo JE, Norville JE, Church GM. 2013 RNA-guided human genome engineering via Cas9. Science 339, 823–826. (doi:10.1126/science.1232033) PMC3712628 23287722

[24] Cong L *et al.* 2013 Multiplex genome engineering using CRISPR/Cas systems. Science 339, 819–823. (doi:10.1126/science.1231143) PMC3795411 23287718

[25] Chen JS , Ma E, Harrington LB, Da Costa M, Tian X, Palefsky JM, Doudna JA. 2018 CRISPR-Cas12a target binding unleashes indiscriminate single-stranded DNase activity. Science 360, 436–439. (doi:10.1126/science.aar6245) PMC6628903 29449511

[26] Gootenberg JS *et al.* 2017 Nucleic acid detection with CRISPR-Cas13a/C2c2. Science 356, 438–442. (doi:10.1126/science.aam9321) PMC5526198 28408723

[27] Patchsung M *et al.* 2020 Clinical validation of a Cas13-based assay for the detection of SARS-CoV-2 RNA. Nat. Biomed. Engineer. 4, 1140–1149. (doi:10.1038/s41551-020-00603-x) 32848209

[28] Lee RA *et al.* 2020 Ultrasensitive CRISPR-based diagnostic for field-applicable detection of *Plasmodium* species in symptomatic and asymptomatic malaria. Proc. Natl Acad. Sci. USA 117, 25 722–25 731. (doi:10.1073/pnas.2010196117) PMC7568265 32958655

[29] Zahra A , Shahid A, Shamim A, Khan SH, Arshad MI. 2023 The SHERLOCK platform: an insight into advances in viral disease diagnosis. Mol. Biotechnol. 65, 699–714. (doi:10.1007/s12033-022-00625-7) PMC9735230 36494593

[30] Kellner MJ , Koob JG, Gootenberg JS, Abudayyeh OO, Zhang F. 2019 SHERLOCK: nucleic acid detection with CRISPR nucleases. Nat. Protoc. 14, 2986–3012. (doi:10.1038/s41596-019-0210-2) PMC6956564 31548639

[31] Siddle HV *et al.* 2013 Reversible epigenetic down-regulation of MHC molecules by devil facial tumour disease illustrates immune escape by a contagious cancer. Proc. Natl Acad. Sci. USA 110, 5103–5108. (doi:10.1073/pnas.1219920110) PMC3612627 23479617

[32] Murchison EP *et al.* 2012 Genome sequencing and analysis of the Tasmanian devil and its transmissible cancer. Cell 148, 780–791. (doi:10.1016/j.cell.2011.11.065) PMC3281993 22341448

[33] Sherlock Biosciences, Inc. 2022 Sherlock CRISPR SARS-CoV-2 kit: instructions for use. FDA-120-0048,. Available from: https://www.fda.gov/media/137746/download.

[34] Schönbichler A *et al.* 2025 Tyrosine kinase targeting uncovers oncogenic pathway plasticity in Tasmanian devil transmissible cancers. EMBO J. 45, 1426–1459. (doi:10.1038/s44318-025-00603-0) PMC12953634 41184587

[35] Maskell KG , Schönbichler A, Flies AS, Patchett AL. 2025 Differentially expressed growth factors and cytokines drive phenotypic changes in transmissible cancers. Discov. Immunol. 4, 1. (doi:10.1093/discim/kyaf011) PMC12314367 40755840

[36] Kayigwe AN , Darby J M., Lyons AB, Patchett A L., Lisowski L, Liu G-S, Flies A S.. 2022 A human adenovirus encoding IFN-γ can transduce Tasmanian devil facial tumour cells and upregulate MHC-I. J. Gen. Virol. 103, 11. (doi:10.1099/jgv.0.001812) 36382885

[37] Flies AS *et al.* 2020 An oral bait vaccination approach for the Tasmanian devil facial tumor diseases. Expert Rev. Vaccines 19, 1–10. (doi:10.1080/14760584.2020.1711058) 31971036

[38] Espejo C , Wilson R, Willms E, Ruiz-Aravena M, Pye RJ, Jones ME, Hill AF, Woods GM, Lyons AB. 2021 Extracellular vesicle proteomes of two transmissible cancers of Tasmanian devils reveal tenascin-C as a serum-based differential diagnostic biomarker. Cell. Mol. Life Sci. 78, 7537–7555. (doi:10.1007/s00018-021-03955-y) PMC11073120 34655299

[39] Espejo C *et al.* 2022 Cathelicidin-3 associated with serum extracellular vesicles enables early diagnosis of a transmissible cancer. Front. Immunol. 13, 858 423. (doi:10.3389/fimmu.2022.858423) PMC9004462 35422813

[40] Li J *et al.* 2025 Optimizing two-step and one-step SHERLOCK nucleic acid detection assays and their applications in detecting influenza A and B viruses. Microchem. J. 211, 113 064. (doi:10.1016/j.microc.2025.113064)

[41] Puig H *et al.* 2021 Minimally instrumented SHERLOCK (miSHERLOCK) for CRISPR-based point-of-care diagnosis of SARS-CoV-2 and emerging variants. Sci. Adv. 7, eabh2944. (doi:10.1126/sciadv.abh2944) PMC8346217 34362739

[42] Kruawan A , KC R, Gell DA, Darby JM, Zhu W, Ong CEB, Fairfax KA, Flies A. 2026 Supplementary material from: Development of a point- of- care field test for Tasmanian devil facial tumour disease. Figshare. (doi:10.6084/m9.figshare.c.8743971) PMC13750945 43012070

