# Supplementary material for: Development of a point-of-care field test for Tasmanian devil facial tumour disease
Source: R Soc Open Sci. 2026 Nov 11;13(11):260028. doi: 10.1098/rsos.260028 (PMC13750945; doi:10.1098/rsos.260028)
Supplement: Supplementary file 1 [file rsos.260028.zip › rsos.260028/rsos260028_si_002.docx]

**SUPPLEMENTARY METHOD 1**

Development of a point-of-care field test for Tasmanian devil facial tumour disease

**AUTHORS**

Anuk Kruawan^1^, Rajendra K.C.^2^, David A Gell^1,2^, Jocelyn M. Darby^1^, Weizhen Zhu^1^, Chrissie E. B. Ong^1^, Kirsten A. Fairfax^2^, Andrew S. Flies^1^*

**AFFILIATIONS**

^1^Menzies Institute for Medical Research, College of Health and Medicine, University of Tasmania, Hobart, TAS 7000, Australia

^2^School of Medicine, College of Health and Medicine, University of Tasmania, Hobart, TAS 7000, Australia

**CORRESPONDING AUTHOR CONTACT INFORMATION**

*Correspondence: andy.flies@utas.edu.au (A.S. Flies)

Menzies Institute for Medical Research, University of Tasmania

GPO Box 341

Hobart, Tasmania, 7001, Australiat: +61 3 6226 4614

**Expression and purification of LwCas13a**

Rosetta 2(DE3)pLysS Singles competent bacteria pre-transformed with pC013 - Twinstrep-SUMO-huLwCas13a (Addgene #90097) were used as an expression system for LwCas13a. A Luria-Bertani agar (LB) plate (Millipore, #28713-500G-F) containing 100 ug/mL of ampicillin was inoculated with bacteria and incubated overnight at 37 °C. Twenty-five mL of Terrific Broth (TB) medium (Invitrogen^TM^, #22711022) containing 100 µg/mL of ampicillin (Sigma-Aldrich, #A9518) was inoculated with a single bacteria colony and incubated overnight at 37 °C at 300 rpm. Four litres of TB medium containing 100 µg/mL ampicillin was inoculated with 20 mL of starter culture. Once the culture reached an optical density (OD) 0.4–0.6, the culture was allowed to cool to room temperature for 30 minutes. The expression of the gene encoding the Twinstrep-SUMO-huLwCas13a recombinant proteins was induced using 4 mL of 0.5 M Isopropyl ß-D-1-thiogalactopyranoside (IPTG, Sigma-Aldrich, #I6758-1G), and the culture was incubated overnight at 21 °C in a shaker at 200 rpm.

The bacteria were harvested by centrifugation at 5,200 × *g* for 15 minutes at 4 °C. Four mL of supplemented lysis buffer (20 mM Tris-HCl pH 8.0, 500 mM NaCl, 1 mM DTT, 1× cOmplete™ ULTRA protease inhibitor cocktail (Roche, #11873580001), 1 mg/mL lysozyme (Sigma-Aldrich, #L6876-1G), 1,500 units Benzonase (Millipore, #E1014-5KU)) per 1 g pelleted bacteria was used. The mixture was placed on the magnetic stirrer for 30 minutes at 4 °C before sonication on ice at the maximum amplitude with 1 s on and 2 s off for the total of 10 minutes of sonication time. The lysate was cleared by centrifugation at 10,000 rpm (17,222 × *g*) at 4 °C for 1 hour (Thermo Fisher Scientific Fiberlite F14-14 x 50cy rotor).

The clear fraction was decanted into a 250-ml tube and 5 ml Strep-Tactin Superflow plus (Qiagen, #30004) was added to batch bind the recombinant protein. The mixture was incubated for 2 hours at 4 °C with gentle shaking. A 100-mL glass chromatography column was prepared in the meantime by washing twice with cold lysis buffer (20 mM Tris-HCl pH 8.0, 500 mM NaCl, 1 mM DTT (Millipore, #3860-5GM)). Twenty mL of cold lysis buffer was then added to equilibrate the column and then drained immediately before addition of the resin solution. The column was then washed with 25 mL cold lysis buffer for 3 times. Fifteen mL of SUMO protease cleavage solution (lysis buffer, 250 units SUMO protease (Sigma-Aldrich, #SAE0067-2500UN), 22.5 μL TERGITOL™ NP-40 (Sigma-Aldrich, #NP40S-100ML) was added into the washed sample-resin. The SUMO protease cleavage was allowed to proceed overnight at 4 °C on the roller. The column was drained and washed 3 times with 5 mL cold lysis buffer to ensure the complete elution of untagged LwCas13a.

The collected sample was purified using ÄKTA Start protein purification system equipped with a 1 mL Sepharose High Performance (HiTrap SP HP) strong cation exchange column (Cytiva, #17115101) to remove SUMO protease, nucleases, and other unwanted contaminants. The column was prepared by washing with 5 column volumes of buffer B (20 mM Tris-HCl pH 7.5, 5% w/v glycerol (Sigma-Aldrich, #G5516-500ML), 1 mM DTT, 2 M NaCl), followed by 5 column volumes of buffer A (20 mM Tris-HCl pH 7.5, 5% w/v glycerol, 1 mM DTT) with a flow rate of 1 mL/min. The column was equilibrated in 12.5% buffer B (to achieve 250 mM NaCl) with a flow rate of 1 mL/min. The untagged LwCas13a was diluted by two-fold with buffer A to lower the NaCl concentration to 250 mM. The diluted sample was applied to the column, followed by column wash with 5 column volumes 12.5% buffer B, gradient elution with 10 column volumes from 12.5% buffer B to 100% buffer B.

Fractions containing LwCas13a were pooled and concentrated using 50 MWCO 15-mL spin filter (4,000 × *g* for 15 min). The buffer was exchanged by adding 15 mL protein storage buffer (50 mM Tris-HCl pH 7.5, 600 mM NaCl, 5% v/v glycerol, 2 mM DTT) to the same spin filter and centrifuged at 4,000 × *g* for 15 min at 4 °C. The purified LwCas13a was diluted to 63.3 µg/mL in protein storage buffer for use with CRISPR detection step. Protein fractions from each stage of purification were analysed using sodium dodecyl-sulfate polyacrylamide gel electrophoresis (SDS-PAGE) to ensure the successful expression and purification. Briefly, 4 μL 10X Bolt™ sample reducing agent (Thermo Fisher Scientific, #B0009), 10 µL 4X Bolt™ LDS Sample Buffer (Thermo Fisher Scientific, #B0007), and 16 µL UltraPure™ water (Invitrogen™, #10977015) were added to 10 µl of samples before heated at 95 °C for 5 minutes then incubated on ice for 2 minutes. Thirty µL of samples were loaded into Bolt™ 4‐12% Bis-Tris Plus Mini Protein Gels (Thermo Fisher Scientific, #NW04120BOX) in 1x Bolt™ MES SDS running buffer (Thermo Fisher Scientific, #B0002). The gel was run at 200 V for approximately 20 minutes. The gel was stained for total protein using GelCode™ Blue Safe Protein Stain (Thermo Fisher Scientific, #24594) according to the manufacturer’s protocol before being visualised (**Supplementary Figure 1**).
